# Neurological diagnoses in children suspected of developmental coordination disorder: DCD or not DCD, that is the question

**DOI:** 10.1111/dmcn.16470

**Published:** 2025-08-22

**Authors:** Jill G. Zwicker

**Affiliations:** ^1^ Department of Occupational Science & Occupational Therapy University of British Columbia Vancouver Canada

## Abstract

**This commentary is on the original article by Garofalo et al. on pages** 251–262 **of this issue.**

The Diagnostic and Statistical Manual of Mental Disorders, Fifth Edition, Text Revision^1^ outlines four diagnostic criteria for developmental coordination disorder (DCD): (A) Acquisition and execution of coordinated motor skills are substantially below expectations for a child's chronological age and opportunities for motor skill learning. Difficulties may include ‘clumsy’, slow, or inaccurate motor skill performance. (B) Motor skills deficit significantly and persistently interferes with activities of daily living appropriate for age, and impacts school‐work, prevocational and vocational activities, leisure, and play. (C) Onset of symptoms in the early developmental period. (D) Motor skills deficit is not better explained by intellectual disability, visual impairment, or a neurological condition affecting movement.

International clinical practice guidelines^2^ recommend that Criterion A is measured with a standardized assessment of motor skills, such as the Movement Assessment Battery for Children, Second Edition (MABC‐2), typically administered by an occupational or physical therapist.^3^ In the study by Garofalo et al.,^4^ only 62% of the sample (31/50) had an MABC‐2 assessment (administered by a pediatric rehabilitation doctor). While they reported that most patients were assessed by the rehabilitation team (49/50), the other team members' roles and involvement in informing the diagnosis was not described. Criterion B was assessed by the physician via parent interview and not quantified with the recommended DCD Questionnaire.^2^, ^3^ Measurement of Criterion C was not explicitly discussed but could be inferred from the age of onset of first symptoms. The main strength of the study by Garofalo et al. is the thoroughness with which the authors assessed Criterion D; children underwent a neurological examination, and some received further testing (e.g. magnetic resonance imaging, laboratory tests, neurophysiological tests, genetic testing, IQ testing). This paper provides a valuable addition to the field by documenting potential alternate diagnoses to DCD that should be considered.

One concern about this study is that the authors suggest that movement disorder features (e.g. ataxia, dystonia, chorea) can be present in DCD;^4^ however, hard neurological signs usually preclude a DCD diagnosis.^3^ Thus, it is unclear if this finding is specific to the diagnostic practices at their centre, which may or may not be accurate. Table S1 provides further concern regarding diagnostic practices for the 15 children who were ultimately given a DCD diagnosis. It is unclear how these children ‘met’ the criteria for DCD, other than the physician ruling out suspected conditions. In these cases, the diagnosis appeared to be made without assessment or consultation with other team members (e.g. occupational or physical therapist). A team approach to diagnosis is considered best practice.^2^, ^3^

An important message from Garofalo et al.^4^ is that the phenotypic presentation of DCD may not be DCD—almost 40% of cases in this study. The authors used retrospective data from one clinical setting (pediatric neurology clinic) to investigate whether initial phenotypical assessment predicted diagnostic outcome in 50 children potentially fulfilling criteria for DCD. The authors found that 62% (31/50) of patients ultimately received a DCD diagnosis, whereas 38% (19/50) received an alternate diagnosis, with 11 of these cases having an underlying genetic etiology. Concordance between the initial suspected diagnosis and reported diagnosis was 40% (20/50), with 16 true positives and four false positives for DCD. Discordant diagnosis was reported in 60% of cases (30/50), with equal distribution between false negative and false positive DCD diagnoses.

The authors concluded that the initial neurological phenotypical assessment is insufficiently predictive of the diagnostic outcome. This finding reinforces the importance of a thorough medical examination and ruling out other potential conditions before giving a DCD diagnosis. Their findings should also make us question whether research samples of children who phenotypically present as DCD are really DCD if they have not been medically investigated. To further advance the field, we need to ask: DCD or not DCD, that is the question.

## Data Availability

Not required.
